# Corona enhancement combined with microvascular invasion for prognosis prediction of macrotrabecular-massive hepatocellular carcinoma subtype

**DOI:** 10.3389/fonc.2023.1138848

**Published:** 2023-02-20

**Authors:** Lili Yang, Meng Wang, Yanyan Zhu, Jiahui Zhang, Junhan Pan, Yanci Zhao, Ke Sun, Feng Chen

**Affiliations:** ^1^ Department of Radiology, The First Affiliated Hospital, Zhejiang University School of Medicine, Hangzhou, Zhejiang, China; ^2^ Department of Pathology, The First Affiliated Hospital, Zhejiang University School of Medicine, Hangzhou, Zhejiang, China; ^3^ Department of Radiology, Third People’s Hospital of Hangzhou, Hangzhou, Zhejiang, China; ^4^ Key Laboratory of Medical Molecular Imaging of Zhejiang Province, Hangzhou, Zhejiang, China

**Keywords:** Magnetic resonance imaging (MRI), hepatocellular carcinoma, prognosis, survival, nomogram

## Abstract

**Objectives:**

The macrotrabecular-massive (MTM) subtype of hepatocellular carcinoma (HCC) is aggressive and associated with an unfavorable prognosis. This study aimed to characterize MTM-HCC features based on contrast−enhanced MRI and to evaluate the prognosis of imaging characteristics combined with pathology for predicting early recurrence and overall survival after surgery.

**Methods:**

This retrospective study included 123 patients with HCC that underwent preoperative contrast−enhanced MRI and surgery, between July 2020 and October 2021. Multivariable logistic regression was performed to investigate factors associated with MTM-HCC. Predictors of early recurrence were determined with a Cox proportional hazards model and validated in a separate retrospective cohort.

**Results:**

The primary cohort included 53 patients with MTM-HCC (median age 59 years; 46 male and 7 females; median BMI 23.5 kg/m2) and 70 subjects with non-MTM HCC (median age 61.5 years; 55 male and 15 females; median BMI 22.6 kg/m2) (All *P*>0.05). The multivariate analysis identified corona enhancement (odds ratio [OR]=2.52, 95% CI: 1.02–6.24; *P*=0.045) as an independent predictor of the MTM-HCC subtype. The multiple Cox regression analysis identified corona enhancement (hazard ratio [HR]=2.56, 95% CI: 1.08–6.08; *P*=0.033) and MVI (HR=2.45, 95% CI: 1.40–4.30; *P*=0.002) as independent predictors of early recurrence (area under the curve=0.790, *P*<0.001). The prognostic significance of these markers was confirmed by comparing results in the validation cohort to those from the primary cohort. Corona enhancement combined with MVI was significantly associated with poor outcomes after surgery.

**Conclusions:**

A nomogram for predicting early recurrence based on corona enhancement and MVI could be used to characterize patients with MTM-HCC and predict their prognosis for early recurrence and overall survival after surgery.

## Introduction

Hepatocellular carcinoma (HCC) is a malignant tumor with poor overall outcomes and a high incidence of postoperative recurrence (1). The highly heterogeneous nature of HCC makes it difficult to make accurate assessments of the recurrence risk and develop appropriate interventions. A new subgroup of HCC, termed macrotrabecular-massive HCC (MTM-HCC) was proposed in the fifth edition of WHO Classification of digestive tumors (2). MTM-HCC is a highly aggressive phenotype with a poor prognosis, according to genetic alterations and molecular features (3, 4). MTM-HCC is suspected when the trabeculae are more than 6 cells thick, and they account for more than 50% of the entire tumor area (5). Prior studies suggested that the hallmark of MTM-HCC is angiogenesis activation, and the poor prognosis is due to frequent macrovascular and/or microvascular invasion, and satellite nodules (5, 6).

Although the MTM subtype can only be confirmed with histopathology, contemporary guidelines do not recommend routine biopsies for hepatic lesions (7, 8). The Liver Imaging Reporting and Data System (LI-RADS), which shows more than 90% accuracy in diagnosing HCC lesions >2 cm, plays a crucial role in HCC management (9, 10). Some studies demonstrated that an arterial phase enhancement pattern in MRI imaging was independently associated with both early and overall tumor recurrence (10, 11). A CT study demonstrated that preoperative imaging findings of intratumor necrosis and hemorrhage were independent predictors of the MTM subtype (12). Similarly, MRI studies showed that, in primary HCC, contrast−enhanced MRI evidence of substantial necrosis combined with intratumor fat, necrosis alone, or severe ischemia could predict MTM-HCC (13, 14). However, those predictive imaging features were inconstant and their prognostic significance was rarely validated in MRI studies.

Therefore, the present study aimed to investigate prognostic features of the MTM-HCC subtype with dynamic contrast-enhanced MRI in patients with primary HCC that underwent a hepatic resection or transplantation. Then, we validated the prognostic value of those features for predicting clinical outcomes after surgery.

## Materials and Methods

### Patients

This retrospective study was approved by the hospital Institutional Review Board. The requirement for written informed consent from patients was waived.

**Figure 1:**
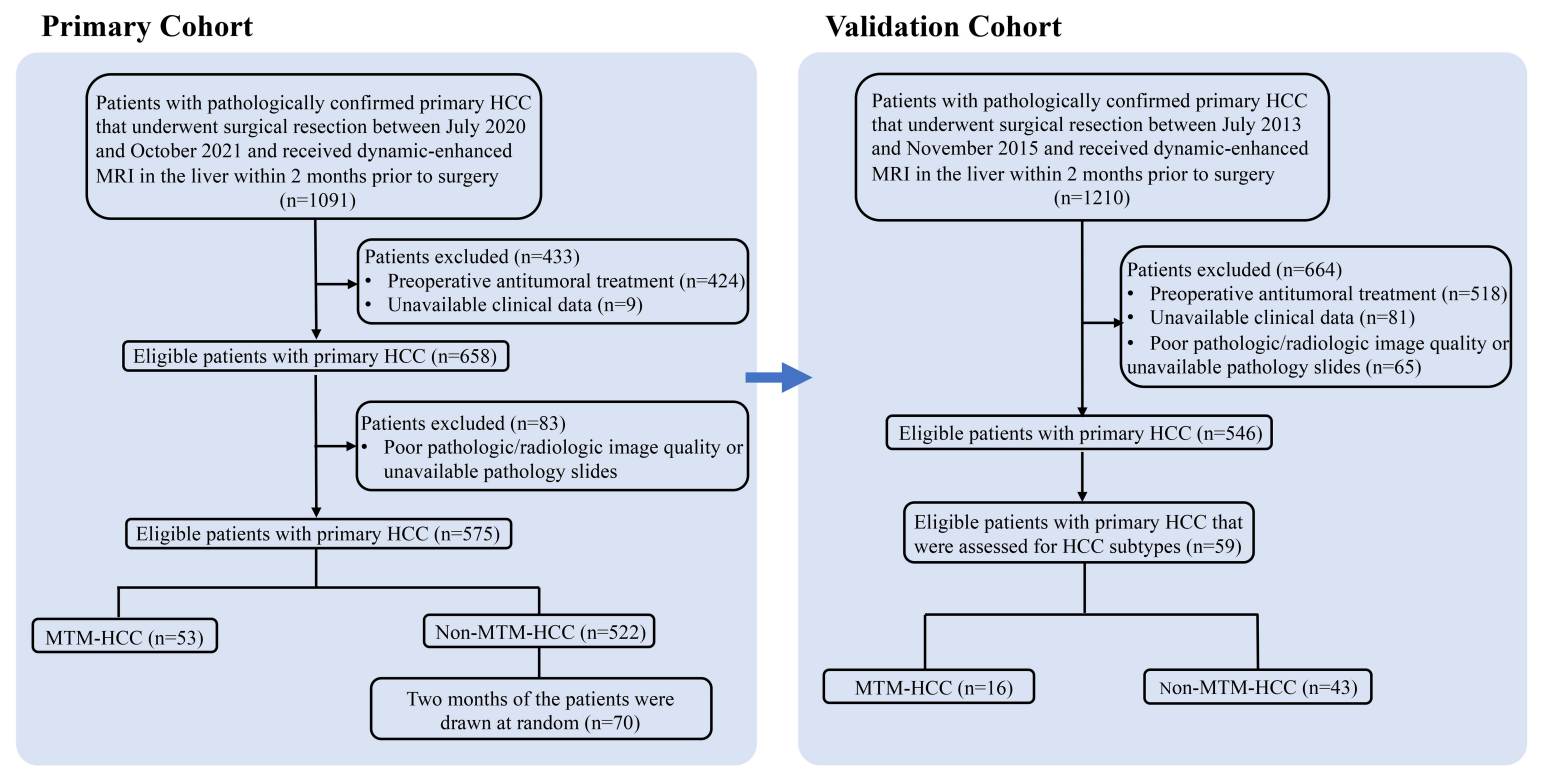
Flow chart shows the patient selection procedure. HCC, hepatocellular carcinoma; MTM, macrotrabecular-massive subtype.

Initially, for the primary cohort, 1091 patients were identified that had undergone a surgical resection or liver transplant for primary HCC between July 2020 and October 2021 at our tertiary care hospital. Patients were included when they had undergone dynamic-enhanced MRI in the liver within 2 months prior to surgery. Exclusion criteria were: (a) preoperative antitumoral treatment; (b) unavailable clinical data; (c) poor quality radiologic or pathologic images; and (d) no pathology slides available for review. Then, 53 patients with a pathologic diagnosis of MTM-HCC and 522 patients with non-MTM HCC were identified. According to the data size of the MTM-HCC group, two months of patients (a total of 70 non-MTM HCC patients) were drawn randomly from the non-MTM HCC group as a control. Finally, 53 patients with MTM-HCC and 70 patients with non-MTM HCC were included (**Figure 1**). The validation cohort comprised other patients that underwent a surgical resection for primary HCC from July 2013 to November 2015 at the same center (15).

Clinical data were retrospectively collected by reviewing electronic medical records. We collected data on patient demographics and survival; the etiology of chronic liver disease (hepatitis B virus; hepatitis C virus; chronic alcohol consumption; family cancer history; preoperative serum levels of aspartate transaminase, alanine transaminase, albumin, serum ferritin, creatinine, platelets, total bilirubin, and γ-glutamyl transpeptidase; the prothrombin time; alpha-fetoprotein (AFP), carbohydrate antigen 125 (CA125), carbohydrate antigen 19-9 (CA199), and carcinoembryonic antigen (CEA); and the Barcelona Clinic Liver Cancer stage.

### Outcome measurements

Patients in this study underwent routine clinical and radiologic follow-ups every 3 to 6 months within the first 2 years after surgery. Radiologic follow-ups included ultrasonography, helical dynamic CT, or MRI. Early HCC recurrence was defined as a recurrence within the first 1 year after surgery (16, 17), regardless of location, based on imaging findings. Patients without recurrence during the follow-up period were censored at the last visit.

### MRI examination

MRI images were acquired with a 3.0 Tesla scanner (primary cohort: Signa HDxt or Discovery MR750; validation cohort: Signa HDxt, both from GE, Waukesha, WI, USA). Detailed MRI parameters are provided in supplemental Appendix S1. Dynamic contrast-enhanced scans were collected in the arterial phase (AP), portal venous phase (PVP), and equilibration phase (EP) at 14-20 s, 45-60 s, and 150-180 s, respectively, after injecting 0.1 mmol/kg Gd-DTPA.

### Qualitative MRI analysis

Preoperative images were retrieved from a picture archiving and communication system and reviewed independently by two faculty abdominal radiologists with at least 10 (Author A) and 9 years (Author B) of experience. Inter-reviewer disagreements were resolved by consensus with a third observer (Author C, 31 years of experience). The three readers were aware of the HCC diagnosis, but were blinded to clinical history and histopathologic findings, including the MTM-HCC subtype. When more than one lesion was present, the largest was selected for analysis in both imaging and pathology. According to the principles underlying the LI-RADS categorization, the following imaging features were used to categorize patients at high risk of HCC: (a) the largest diameter of the main lesion; (b) the number of segments involved; (c) irregular tumor margins; (d) substantial necrosis, defined as a central area of high-signal intensity on fat-suppressed turbo spin-echo T2-weighted images; (e) hemorrhagic component; (f) fat component; (g) rim arterial phase hyperenhancement (APHE); (h) APHE; (i) corona enhancement (i.e., periobservational enhancement in late arterial phase or early PVP attributable to venous drainage from tumor)defined in LI-RADS 2018 (18); in our study, late AP or early PVP defined as the following: Hepatic artery and branches are fully enhanced, and portal vein is partially or fully enhanced before liver parenchyma reach peak enhancement; (j) non-peripheral washout; (k) enhancing capsule; and (l) tumor in vein.

### Histopathologic examination

All the histologic slides were reviewed by an abdominal pathologist with 12 years of experience in liver pathology. The following pathological features were recorded: a. the presence of the MTM subtype, defined as a predominant trabeculae more than 6 cells thick and that occupied more than 50% of the entire tumor; b. tumor differentiation (according to the Edmonson Steiner grade); c. the presence of macrovascular invasion or microvascular invasion (MVI, a pathological criterion defined by tumor emboli in portal radicle veins or vascular spaces lined by endothelial cells in the peritumoral liver (19); MVIs were graded as M0: no MVI (MVI negative), M1: MVI of < 5 and at ≤ 1 cm away from the adjacent liver tissues, and M2: MVI of > 5 or at > 1 cm away from the adjacent liver tissues. In this study, M1 or M2 stands for MVI positive); d. the presence of satellite nodules; and e. the presence/absence of a capsule (i.e., absent, incomplete, or complete).

### Statistical analysis

Continuous variables are presented as the median and interquartile range (IQR). Categorical variables are expressed as numbers and percentages. Continuous variables were compared with the Mann-Whitney U test or Student t-test. Categorical variables were compared with the Chi-square test or Fisher’s exact test. Univariable and multivariable logistic regression analyses were performed to identify independent risk factors for the MTM-HCC subtype. Univariable and multivariable Cox regression analyses were performed to identify predictors of early HCC recurrence. Receiver operating characteristic (ROC) curves were constructed to predict early recurrence. Survival was analyzed with the Kaplan-Meier method and log-rank test. Statistical analyses were performed with SPSS (version 22.0) and R software (version 3.5.3). A *P*<0.05 (two-tailed) was considered statistically significant.

## Results

### Clinical and pathologic patient characteristics

The primary cohort included 123 subjects; 53 with MTM-HCC and 70 with non-MTM HCC. The validation cohort included 59 subjects; 16 with MTM-HCC and 43 with non-MTM HCC (**Figure 1**). A comparison of clinical and pathologic variables between the two cohorts showed that the main tumor size and the CA199 level were significantly lower, and the CEA level was significantly higher, in the primary cohort than in the validation cohort. The other variables were sufficiently matched between cohorts **(** **Supplemental Table 1**). However, cirrhosis occurred more frequently in the MTM-HCC group of the primary cohort, than in the MTM -HCC group of the validation cohort.

Clinical and pathologic characteristics of the primary cohort were compared between MTM-HCC and non-MTM HCC groups (**Tables 1**, **2**). The median age, sex ratio, and median BMI were comparable between the MTM-HCC group and the non-MTM HCC group (All *P*>0.05; **Table 1**). The proportion of patients with Barcelona Clinic Liver Cancer Stage B or C disease was somewhat higher in the MTM-HCC (37.7%) than in the non-MTM HCC group (22.9%; borderline *P*=0.07), but other characteristics were not significantly different between the two groups (**Table 1**). At the pathologic level, the MTM- HCC group had significantly more instances of multiple (2 or ≥3) tumors (15.1% vs. 2.8%; *P*=0.015), Edmondson-Steiner grade III or IV tumors (79.5% vs. 63.9%; *P*=0.05), and stage 1 or 2 MVI (49% vs. 27.1%; *P*=0.009), compared to the non-MTM HCC group (**Table 2**).

**Table 1:** Clinical and pathologic characteristics of patients with macrotrabecular-massive subtype of HCC.

Characteristic	MTM-HCC	Non MTM-HCC	*P*
	(n=53)	(n=70)	
Age, years	59 (49.5-65.5)	61.5 (51.5-67.0)	0.594
Sex, male/female	46/7	55/15	0.239
BMI, kg/m^2^	23.5 (21.3-25.6)	22.6 (21.2-24.7)	0.477
^*^Chronic alcohol consumption	7 (15.6)	12 (30.8)	0.096
^*^Family cancer history	11 (23.9)	5 (12.8)	0.192
HBV infection	43 (81.1)	54 (77.1)	0.592
HCV infection	1 (1.9)	3 (4.3)	0.818
AFP, ng/ml	50 (4.70-563.3)	26.9 (4.1-369.4)	0.445
CEA, ng/ml	2.7 (1.7-4.2)	2.7 (1.9-3.9)	0.473
^†^CA125, U/ml	12.6 (9.1-20.1)	11.4 (8.0-15.1)	0.118
^†^CA199, U/ml	5.9 (3.3-13.0)	7.2 (3.1-16.8)	0.375
Albumin, g/L	43.5 (41.3-46.7)	43.6 (40.1-45.8)	0.563
^†^SF, ng/ml	202.4 (123.5-319.1)	223.9 (131.8-331.6)	0.394
Cretinine, μmol/L	76.0 (66.5-83.5)	75.0 (65.8-84.0)	0.806
AST, U/L	29.0 (22.0-39.0)	28.5 (22.0-36.3)	0.781
ALT, U/L	27.0 (18.0-40.0)	27.5 (18.8-42.8)	0.850
ALP, U/L	83.0 (67.0-105.0)	87.0 (68.8-113.3)	0.358
TB, μmol/L	14.7 (10.9-19.2)	14.4 (10.6-20.3)	0.330
GGT, U/L	42.0 (24.0-66.0)	47.0 (24.8-81.8)	0.601
Platelet count, 10^9^/L	161.0 (120.5-210.5)	142.0 (97.0-202.0)	0.658
PT, second	12.0 (11.3-13.1)	12.3 (11.6-12.8)	0.715
BCLC stage B-C	20 (37.7)	16 (22.9)	0.073

^†^Available number of Carbohydrate antigens 125, Carbohydrate antigens 19-9 and Serum ferritin is 121, 122 and 121, independently.

Variables expressed as median (interquartile range) or number of patients (percentage). HCC, Hepatocellular Carcinoma; HBV, Hepatitis B virus; HCV, Hepatitis C virus; AFP, Alpha-fetoprotein; CEA, Carcinoembryonic antigen; CA125, Carbohydrate antigens 125; CA199, Carbohydrate antigens 19-9; SF, serum ferritin; AST, Aspartate aminotransferase; ALT, Alanine aminotransferase; TB, Total bilirubin; GGT, γ-glutamyl transpeptidase; PT, prothrombin time.

^*^Available number of History of alcohol intake and family cancer history is 84 and 85, independently.

**Table 2:** Pathologic characteristics of patients with hepatocellular carcinoma in the primary cohort according to macrotrabecular-massive subtype.

Characteristic	MTM-HCC	Non MTM-HCC	*P*
	(n=53)	(n=70)	
Tumor number			**0.015**
1	45 (84.9)	68 (97.1)	
2	6 (11.3)	1 (1.4)	
≥ 3	2 (3.8)	1 (1.4)	
Absent or incomplete capsule	52 (98.1)	67 (95.7)	0.818
Edmonson-Steiner grade			**0.050**
I-II	11 (20.5)	26 (37.1)	
III-IV	42 (79.5)	44 (63.9)	
Satellite nodules	6 (11.3)	11 (15.7)	0.484
Macrovascular invasion	3 (5.7)	1 (1.4)	0.425
MVI			**0.009**
0	27 (50.9)	51 (72.9)	
1	19 (35.8)	14 (20)	
2	7 (13.2)	5 (7.1)	
TMN stage			0.168
I	30 (56.6)	47 (67.1)	
II	17 (32.1)	20 (28.6)	
III	6 (11.3)	3 (4.3)	
IV	0	0	
Cirrhosis	30 (56.6)	43 (61.4)	0.590

Variables are expressed as numbers of patients (percentages).

MVI, microvascular invasion.while p value ≤0.05, it was shown in bold.

### MRI findings for the MTM-HCC Subtype

**Figure 2:**
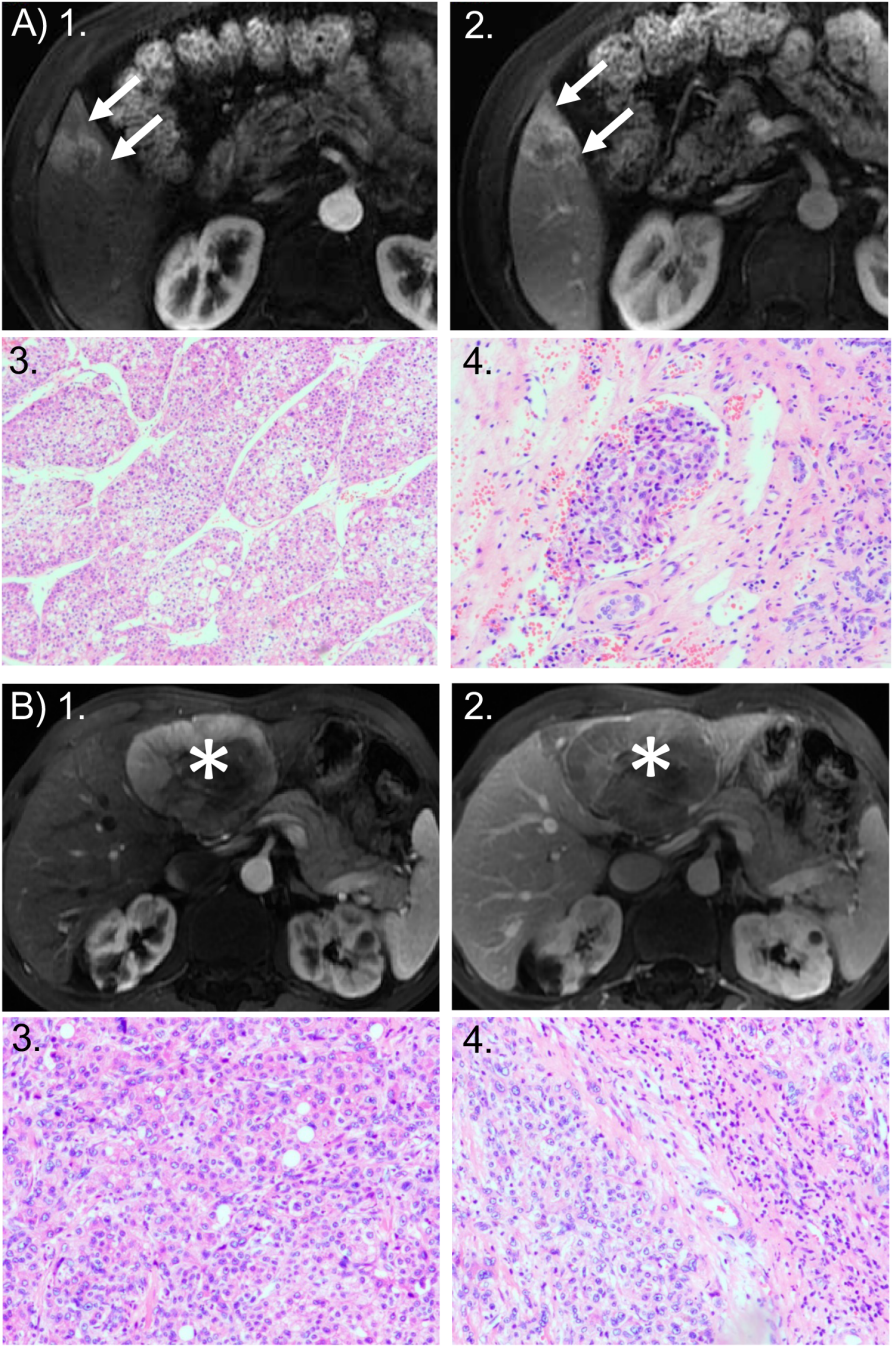
Representative cases of MTM and non-MTM subtype of HCC. **(A)** MTM HCC: 49-year-old woman with a 2.8-cm HCC tumor in hepatic segment V. HCC was the macrotrabecular-massive (MTM) subtype and showed microvascular invasion (MVI). Axial MRI scans in the: (1) arterial phase and (2) portal venous phase show the tumor with corona enhancement: a periobservational region of irregular bright enhancement (*arrow*). Photomicrographs show the predominant MTM appearance: (3) the trabeculae are more than 6 cells thick, and they account for more than 50% of the entire tumor area (hematoxylin-eosin stain; original magnification, ×50); and (4) the presence of clusters of tumor cells covered by vascular endothelial cells in the peritumoral liver (hematoxylin-eosin stain; original magnification, ×100). Recurrence occurred 11 months after surgical resection. **(B)** non-MTM HCC: 67-year-old man with 8.1-cm non-MTM HCC. Axial arterial phase MRIs in the: (1) arterial phase and (2) portal venous phase show heterogeneous hyperenhancement in the mass (asterisks) with a clear border, but no corona enhancement. Photomicrographs show: (3) a microtrabecular pattern; and (4) the absence of MVI. Both images are hematoxylin-eosin stained sections; original magnification, ×100. Recurrence did not occur within 2 years after surgical resection.

The MTM-HCC group more frequently displayed irregular tumor margins (81.1% vs. 64.3%; *P*=0.04), substantial necrosis (39.6% vs. 20.0%; *P*=0.017), a hemorrhagic component (28.3% vs. 10.0%; *P*=0.009), and corona enhancement (34.0% vs. 17.1%; *P*=0.031) compared to the non-MTM HCC group (**Table 3**). The MTM-HCC and non-MTM HCC groups showed no other significant differences, including the LI-RADS categories (**Table 3**). Representative cases of MVI^+^CoroEh^+^ and MVI^−^CoroEh^−^ are shown in **Figures 2A, B**.

**Table 3:** Mri findings of hcc lesions in the primary cohort according to macrotrabecular-massive subtype.

MRI Findings	MTM-HCC	Non MTM-HCC	*P*
	(n=53)	(n=70)	
Main tumor size (cm)	3.7 (2.1-5.6)	3.4 (2.2-5.3)	0.290
Segments involvement			0.153
1	33 (62.3)	52 (74.3)	
2	17 (32.0)	14 (20)	
≥ 3	3 (5.67)	4 (5.71)	
Irregular tumor margin	43 (81.1)	45 (64.3)	**0.040**
Substantial necrosis	21 (39.6)	14 (20.0)	**0.017**
Hemorrhagic component	15 (28.3)	7 (10)	**0.009**
Fat component	15 (28.3)	10 (14.3)	0.056
Rim APHE	5 (4.1)	2 (1.6)	0.244
Non-Rim APHE	46 (86.8)	65 (92.9)	0.262
Corona enhancement	18 (34)	12 (17.1)	**0.031**
Non-peripheral washout	49 (92.5)	62 (88.6)	0.473
Enhancing capsule	35 (66)	41 (58.6)	0.399
Tumor in vein	8 (15.1)	5 (7.1)	0.155
*LI-RADS version 2018 category			0.529
LR-III	3 (5.9)	0 (0)	
LR-IV	3 (5.9)	6 (9.2)	
LR-V	35 (68.6)	52 (80)	
LR-M	3 (5.9)	2 (3.1)	
LR-TIV	7 (13.7)	5 (7.7)	

Variables are expressed as median (interquartile range) or number of patients(percentage).

HCC, Hepatocellular Carcinoma; APHE, arterial phase hyperenhancement; LI-RADS, Liver Imaging Reporting and Data System.

*Determined in 116 patients for whom LI-RADS algorithm can be applied.while p value ≤0.05, it was shown in bold.

### Risk factors for the MTM Subtype

The univariable logical regression analysis identified three MRI features that were positively associated with a high risk of MTM-HCC: an irregular tumor margin (odds ratio [OR]=2.39, 95% CI: 1.03–5.56; *P*=0.04), substantial necrosis (OR=2.63, 95% CI: 1.18–5.86; *P*=0.019), hemorrhagic component (OR=3.55, 95% CI: 1.33–9.50; *P*=0.012), and corona enhancement (OR=2.49, 95% CI: 1.07–5.78; *P*=0.034; **Table 4**). However, a multivariate analysis showed that corona enhancement (OR=2.52, 95% CI: 1.02–6.24; *P*=0.045) was the only independent predictor of the MTM-HCC subtype. The other characteristics were not independent predictors of MTM-HCC (**Table 4**).

**Table 4:** Predictors for macrotrabecular-massive subtypeby of hcc in the primary cohort by logistic regression analysis.

Variable	Univariable OR	*P*	Multivariable OR	*P*
Age, years	0.99 (0.96-1.02)	0.590		
Main tumor size (cm)	1.07 (0.94-1.23)	0.316		
Sex (male)	1.79 (0.67-4.77)	0.243		
MRI features				
Multiple segments involvement	1.41 (0.77-2.59)	0.267		
Irregular tumor margin	2.39 (1.03-5.56)	**0.040**	1.98 (0.80-4.87)	0.139
Substantial necrosis	2.63 (1.18-5.86)	**0.019**	1.89 (0.75-4.74)	0.176
Hemorrhagic component	3.55 (1.33-9.50)	**0.012**	2.75 (0.92-8.20)	0.070
Fat component	2.37 (0.97-5.81)	0.060	2.02 (0.77-5.29)	0.154
Rim APHE	3.54 (0.66-19.0)	0.140		
APHE	0.51 (0.15-1.70)	0.268		
Corona enhancement	2.49 (1.07-5.78)	**0.034**	2.52 (1.02-6.24)	**0.045**
Non-peripheral washout	1.58 (0.45-5.56)	0.475		
Enhancing capsule	1.38 (0.67-2.89)	0.399		
Tumor in vein	2.31 (0.71-7.52)	0.164		
LI-RADS version 2018 category	0.53 (0.21-1.36)	0.187		

Data in parentheses are 95% CIs.

HCC, Hepatocellular Carcinoma.while p value ≤0.05, it was shown in bold.

### Development of the combined prediction model for early recurrence

**Figure 3:**
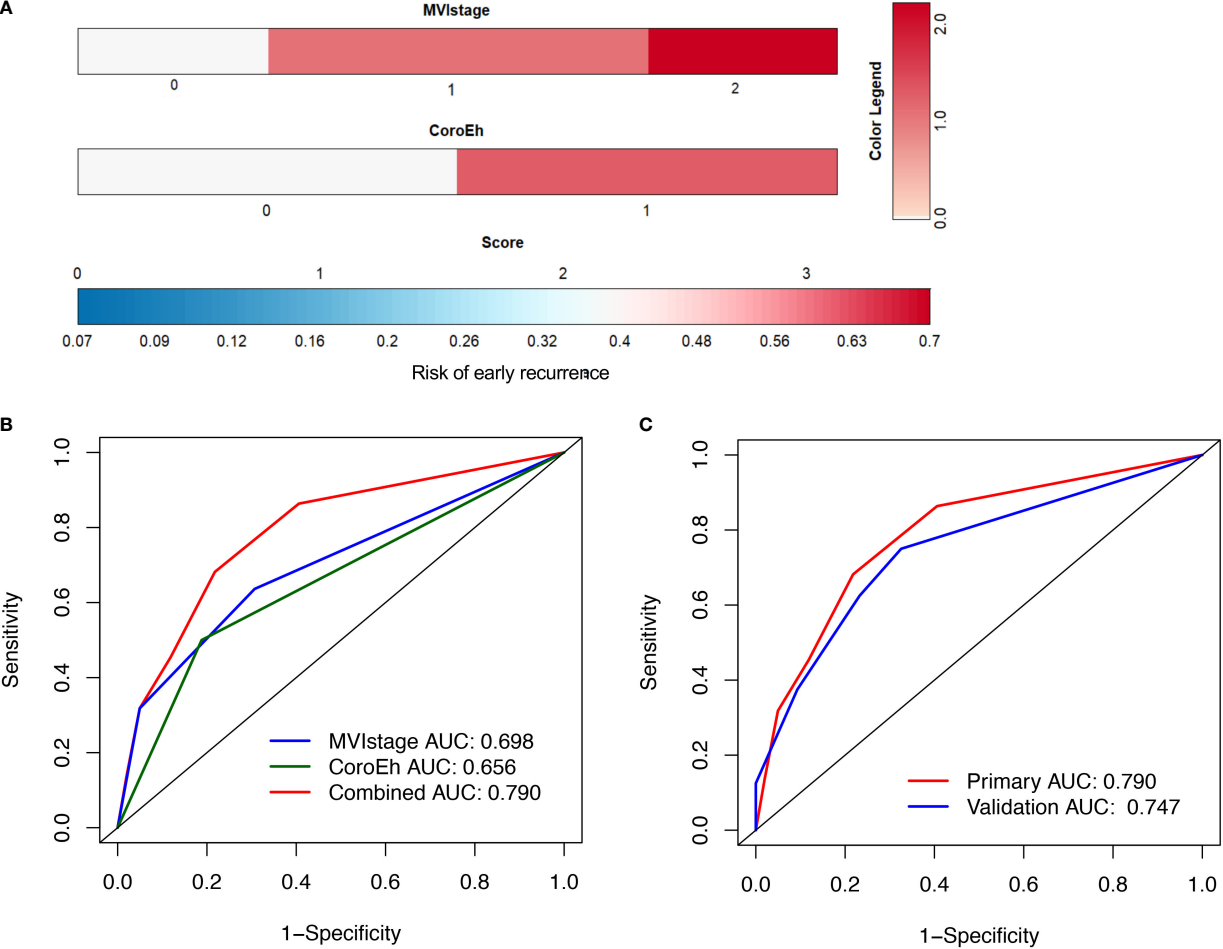
Combined factors for predicting risk of early HCC recurrence. **(A)** nomogram model incorporates the microvascular invasion (MVI) stage and corona enhancement (CoroEh); the sum is used for predicting early recurrence. **(B)** Receiver Operating Characteristic (ROC) curve shows the predictive value of the nomogram model in the primary cohort. The combined model (red line) shows better predictive ability than either single factor. The area under the ROC curve (AUC) of the combined model was 0.790, with 78.2% specificity and 68.2% sensitivity. **(C)** The predictive value of the combined model was comparable in the validation cohort; the AUC was 0.747, with 67.4% specificity, and 75.0% sensitivity.

Cox regression analyses identified several factors that influenced an early HCC recurrence after surgical resection (**Table 5**). A univariable analysis showed that a main tumor size ≥5 cm (hazard ratio [HR]=3.21, 95% CI: 1.39–7.44; *P*=0.006), corona enhancement (HR=3.53, 95% CI: 1.53–8.15; *P*=0.003), the MVI (HR=2.84, 95% CI: 1.65–4.90; *P*<0.001), the Edmonson-Steiner grade (HR=2.29, 95% CI: 1.05–4.97; *P*<0.037), and macrovascular invasion (HR=2.86, 95% CI: 1.23–6.61; *P*<0.014) were associated with early HCC recurrence (**Table 5**). Furthermore, a multivariate analysis showed that corona enhancement (HR=2.56, 95% CI: 1.08–6.08; *P*=0.033) and the MVI (HR=2.45, 95% CI: 1.40–4.30; *P*=0.002) were independent predictors of early HCC recurrence (**Table 5**). Therefore, we selected corona enhancement and MVI to establish a nomogram model for predicting early HCC recurrence (**Figure 3A**). This model provided better predictions of early HCC recurrence than any single factor (**Figure 3B**). For the combined model, the cutoff value derived from the primary cohort was −1.520, and that derived from the validation cohort was −2.181. ROC curve analyses of the combined model showed areas under the curve (AUCs) of 0.790 (95% CI: 0.686, 0.894), for the primary cohort, and 0.747 (95% CI: 0.606, 0.889) for the validation cohort (**Figure 3C**).

**Table 5:** Univariable and Multivariable Cox Regression Analysis for Early Recurrence of Hepatocellular Carcinoma in Primary Cohort after Surgical Resection.

Variable	Univariable HR	*P*	Multivariable HR	*P*
Age, years		0.433		
main tumor size ≥5cm	3.21 (1.39-7.44)	**0.006**		0.056
Sex, male/female		0.590		
MRI features				
multiple segments involvement		0.501		
Irregular tumor margin		0.095		
Substantial necrosis		0.442		
Hemorrhagic component		0.588		
Fat component		0.903		
Rim APHE		0.590		
APHE		0.848		
Corona enhancement	3.53 (1.53-8.15)	**0.003**	2.56 (1.08-6.08)	**0.033**
Non-peripheral washout		0.345		
Enhancing capsule		0.522		
LI-RADS version 2018 category		0.755		
Pathologic Characteristics				
Two or more lesions		0.191		
Absent or incomplete capsule		0.133		
MVI	2.84 (1.65-4.90)	**<0.001**	2.45 (1.40-4.30)	**0.002**
Edmonson-Steiner grade	2.29 (1.05-4.97)	**0.037**		0.085
Macrovascular invasion	2.86 (1.23-6.61)	**0.014**		0.965
Satellite nodules		0.092		
Cirrhosis		0.927		

Data in parentheses are 95% CIs.

APHE, arterial phase hyperenhancement; LI-RADS, Liver Imaging Reporting and Data System; MVI, microvascular invasion.

### Survival analysis for recurrence risk stratification

**Figure 4:**
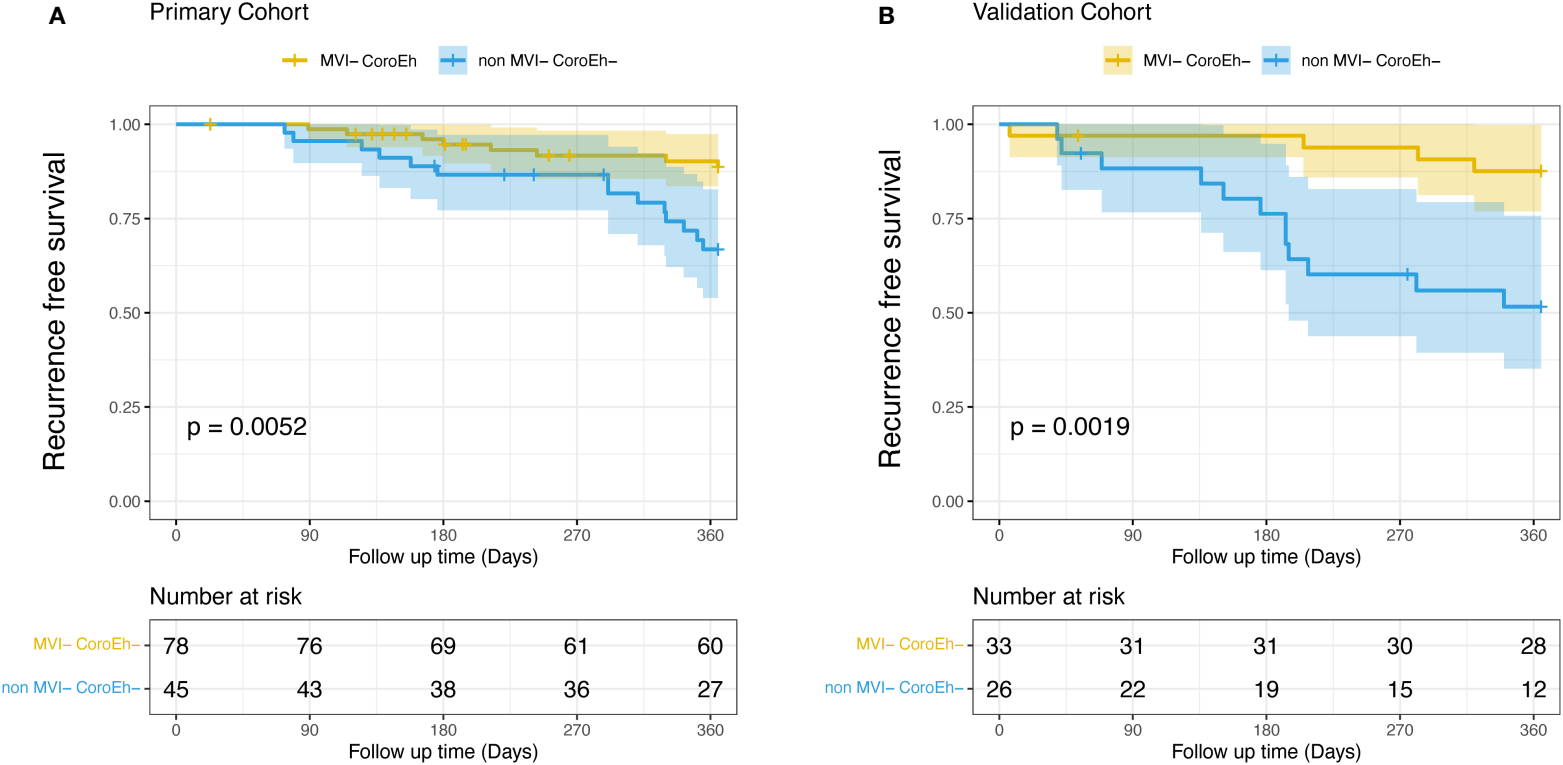
Kaplan-Meier plots show the impact of microvascular invasion (MVI) risk and coronal enhancement (CoroEh) status on early post-surgical recurrence-free survival. Patients without MVI or coronal enhancement (MVI^−^ CoroEh^−^) were compared to the pooled group of patients with MVI and/or coronal enhancement (non-MVI^−^ CoroEh^−^) in **(A)** the primary cohort and **(B)** the validation cohort. *P*-values are based on the log-rank test.

In the primary cohort, early recurrence occurred in 22 (17.9%) of 123 patients, and the median recurrence-free survival was 227 days (range, 73–365 days). We stratified patients into two risk subgroups: one included patients (78/123) with favorable prognoses, with low scores in both MVI and coronal enhancement (MVI^−^CoroEh^−^). The other subgroup included patients (45/123) with a high score in either or both factors (MVI^+^CoroEh^−^; MVI^−^CoroEh^+^; or MVI^+^CoroEh^+^); this group was called the non-MVI^−^CoroEh^−^ group. A Kaplan-Meier survival analysis showed that, compared to the MVI^−^CoroEh^−^ subgroup, the non-MVI^−^CoroEh^−^ subgroup had a higher risk of early recurrence (*P*=0.0052; **Figure 4A**). This difference was also observed in the validation cohort (33/59 MVI^−^CoroEh^−^ vs. 26/59 non-MVI^−^CoroEh^−^, *P*=0.0019; **Figure 4B**).

**Figure 5:**
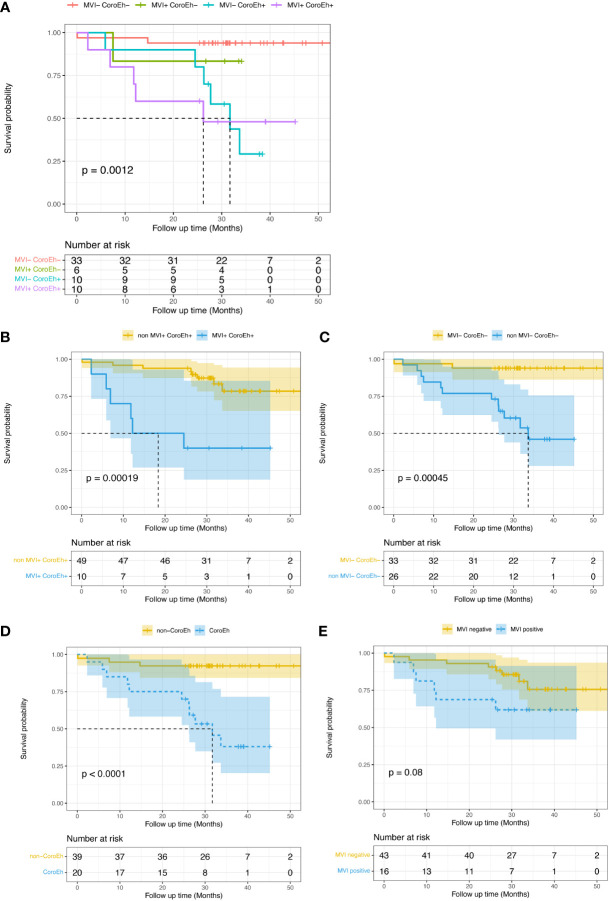
Kaplan-Meier plots show the impacts of macrovascular invasion (MVI) risk and coronal enhancement status (CoroEh) on overall survival (OS) after surgery in the validation cohort. **(A)** OS is compared among four validation subgroups: patients without MVI or CoroEh (MVI^−^ CoroEh^−^) and patients with one or both markers (MVI^+^ CoroEh^−^, MVI^−^ CoroEh^+^, and MVI^+^ CoroEh^+^). OS was significantly associated with the **(B)** non-MVI^+^ CoroEh^+^ (p<0.001) and **(C)** MVI^−^ CoroEh^−^ (*P*<0.001) subgroups. OS was also significantly associated with **(D)** CoroEh status (*P*<0.0001); however, **(E)** MVI was not significantly associated with OS. *P*-values were based on the log-rank test.

Seeing that overall survival (OS) was apparently shorter in the MTM HCC than in the non-MTM HCC patients (*P*<0.0001, Supplemental **Figure 1A**), we furtherly evaluated the prognostic role of the MVI-CoroEh model in OS. We stratified the validation cohort into four significantly different risk subgroups (*P*=0.0012, **Figure 5A**): the MVI^−^CoroEh^−^ subgroup (33/59); the MVI^+^CoroEh^−^ subgroup (6/59); the MVI^−^CoroEh^+^ subgroup (10/59); and the MVI^+^CoroEh^+^ subgroup (10/59). We found that OS was significantly shorter in the MVI^+^CoroEh^+^ subgroup than in the other three groups combined (*P*<0.001, **Figure 5B**). Moreover, the MVI^−^CoroEh^−^ subgroup had a significantly longer OS than the other three groups combined (*P*<0.001, **Figure 5C**). OS was shorter in the CoroEh^+^ subgroups than in the CoroEh^−^ subgroups (*P*<0.0001, **Figure 5D**), but OS was only slightly shorter in the MVI^+^ subgroups than in the MVI^-^ subgroups (*P*=0.08, **Figure 5E**; **Supplemental Figure 1B**).

## Discussion

This study showed that corona enhancement was an independent risk factor for identifying the MTM subtype and for preoperatively predicting early recurrence. Additionally, MVI was found to be an independent pathologic predictor. Furthermore, we built a nomogram that combined these two parameters; this nomogram could predict early HCC recurrence and OS, and its predictive value was confirmed in a validation cohort. Our results may provide insight into the essential relationship between coronal enhancement, MVI, and survival in patients with MTM-HCC. Our findings could serve as a reference for clinicians and surgeons in selecting the most appropriate treatment strategy.

Corona enhancement is an ancillary feature of the LI-RADS that favors malignancy; it is typically observed with high arterial input and high venous output around the tumor (20). To date, few studies have analyzed the sensitivity or specificity of corona enhancement for diagnosing or predicting the clinical outcome of MTM-HCC. The present study showed that corona enhancement was an independent factor for identifying MTM-HCC and an independent preoperative predictor of early HCC recurrence. Based on previous studies, corona enhancement was associated with an increased incidence of micrometastases (21); furthermore, it was both a sensitive predictor of high-grade HCC and associated with poor clinical outcomes after resection (22). Thus, it was not surprising that MTM-HCC showed highly aggressive characteristics and significantly more corona enhancement, compared to non-MTM HCC.

Cell growth, migration, and angiogenesis activation were previously identified as markers of MVI in HCC (23). Accordingly, MVI was identified as an independent postoperative factor for predicting early recurrence and OS in HCC (24). Similarly, in this study, MTM-HCC was associated with significantly higher MVI, compared to non-MTM HCC. Corona enhancement and MVI were positively correlated previously. Lee and colleagues showed that corona enhancement was an imaging biomarker for predicting MVI (25). Rhee et al. showed that corona enhancement was part of a combined identifying factor for MTM-HCC, and MVI was an independent prognostic factor for both early recurrence and poor OS (10). Furthermore, in the present study, corona enhancement and MVI were first demonstrated as separate independent preoperative and postoperative risk factors for identifying MTM-HCC and for predicting prognosis, respectively. The combined model showed AUCs of 0.790 and 0.747 for predicting early recurrence of MTM-HCC in the primary and validation cohorts, respectively. Few prognostic models are available for this HCC subtype; thus, the establishment of a nomogram model for predicting early recurrence should provide improved individualized follow-up strategies for postoperative care (10).

Consistent with previous studies (10, 12, 14), our univariate analysis indicated that the presence of an irregular tumor margin, substantial necrosis, and a hemorrhagic component were significantly different between MTM-HCC and non-MTM HCC groups. However, in the multivariate analysis, none of these factors were identified as independent predictors of MTM-HCC. This result might be explained by the relatively small median lesion sizes and the similarity in median lesion sizes between the MTM (3.7 cm, IQR: 2.1-5.6) and non-MTM (3.4 cm, IQR: 2.2-5.3) groups. Indeed, lesion sizes were larger in studies by Cannella et al. (26) and Feng et al. (12), where intratumor necrosis and hemorrhage were found to be identifying factors for MTM-HCC. In HCC, fast-growing or large tumors have insufficient blood supply in the center, which frequently results in a hypoxic intratumoral microenvironment and creates necrotic vessels that rupture (27). However, our results were consistent with some other studies. For example, a previous multicenter study mentioned that tumor necrosis, to some extent, contributed to a false positive diagnosis of MTM-HCC and that an irregular tumor margin showed no association with MTM-HCC (10). Future studies, based on a larger patient cohort, should take the main tumor size into account, when including patients, to eliminate confounding due to variable tumor sizes.

Previously, high serum AFP concentrations were moderately correlated with tumor size (28). Serum AFP may increase in large tumors, due to an increase in intratumoral cell apoptosis. Conversely, small tumors have lower AFP levels (29). It is conceivable that the relatively small HCC lesions found in our cohort might explain the lack of significant differences in AFP levels and hemorrhagic components between the MTM-HCC and HCC groups (30, 31).

This study had some limitations. First, the retrospective nature of the study introduced a selection bias. Second, patients were included based on surgery; therefore, the results might not be generalizable to all HCCs. Our results should be further validated among patients that are undergoing biopsy. Third, the study had a single-center design with a short follow-up time. Further external validation is needed.

In conclusion, we showed that preoperative multiphase contrast-enhanced MRI features were useful for classifying MTM-HCC. Furthermore, we developed a nomogram of corona enhancement combined with MVI, which showed high sensitivity and specificity in predicting early recurrence and OS. This nomogram could assist clinicians in determining individual follow-up strategies for improved outcomes.

## Author contributions

LY and FC were responsible for the conception of the work. MW and KS reviewed pathological pictures. LY, JP and JZ obtained the clinical data. LY, YZhu and FC reviewed the images. LY, YZhao and FC analyzed the data. LY wrote the manuscript. FC critically revised the manuscript. All authors are accountable for the contents of this work. All authors contributed to the article and approved the submitted version (see green marks in this section).

## Supplementary material

Supplementary Table 1 Clinical and Pathologic Features of Patients with Hepatocellular Carcinoma in the Primary and Validation Cohort. Note: Variables are expressed as median (interquartile range) or number of patients(percentage). Abbreviations: HBV= Hepatitis B virus, AFP= Alpha-fetoprotein, CEA= Carcinoembryonic antigen, CA125= Carbohydrate antigens 125, CA199= Carbohydrate antigens 19-9, SF= serum ferritin. ^*^ Available number of Carbohydrate antigens 125, Carbohydrate antigens 19-9 and Serum ferritin is 121, 122 and 121, independently.

Supplementary Figure 1 Kaplan–Meier overall survival curves of MTM HCC and non-MTM HCC patients(A); different subgroups of MVI risk patients(B).

## References

[1] Marrero JA Kulik LM Sirlin CB Zhu AX Finn RS Abecassis MM. Diagnosis, staging, and management of hepatocellular carcinoma: 2018 practice guidance by the American association for the study of liver diseases: Marrero et al. Hepatology (2018) 68:723–50. doi: 10.1002/hep.29913 29624699

[2] IARC. WHO classification of tumours editorial board. digestive system tumours. 5th ed. Digestive System Tumours. Lyon, France: IARC (2019) p. 229–39.

[3] Calderaro J Ziol M Paradis V Zucman-Rossi J. Molecular and histological correlations in liver cancer. J Hepatol (2019) 71:616–30. doi: 10.1016/j.jhep.2019.06.001 31195064

[4] Calderaro J Couchy G Imbeaud S Amaddeo G Letouzé E Blanc J-F. Histological subtypes of hepatocellular carcinoma are related to gene mutations and molecular tumour classification. J Hepatol (2017) 67:727–38. doi: 10.1016/j.jhep.2017.05.014 28532995

[5] Ziol M Poté N Amaddeo G Laurent A Nault J-C Oberti F. Macrotrabecular-massive hepatocellular carcinoma: A distinctive histological subtype with clinical relevance. Hepatology (2018) 68:103–12. doi: 10.1002/hep.29762 29281854

[6] Tan PS Nakagawa S Goossens N Venkatesh A Huang T Ward SC. Clinicopathological indices to predict hepatocellular carcinoma molecular classification. Liver Int (2016) 36:108–18. doi: 10.1111/liv.12889 PMC4674393 26058462

[7] Galle PR Forner A Llovet JM Mazzaferro V Piscaglia F Raoul J-L. EASL clinical practice guidelines: Management of hepatocellular carcinoma. J Hepatol (2018) 69:182–236. doi: 10.1016/j.jhep.2018.03.019 29628281

[8] Heimbach JK Kulik LM Finn RS Sirlin CB Abecassis MM Roberts LR. AASLD guidelines for the treatment of hepatocellular carcinoma: Heimbach et al. Hepatology (2018) 67:358–80. doi: 10.1002/hep.29086 28130846

[9] American College of radiology. liver imaging reporting and data system . Available at: https://www.acr.org/ClinicalResources/Reporting-and-Data-Systems/LIRADS (Accessed 4 May 2020).

[10] Rhee H Cho E-S Nahm JH Jang M Chung YE Baek S-E. Gadoxetic acid-enhanced MRI of macrotrabecular-massive hepatocellular carcinoma and its prognostic implications. J Hepatol (2021) 74:109–21. doi: 10.1016/j.jhep.2020.08.013 32818570

[11] Mulé S Galletto Pregliasco A Tenenhaus A Kharrat R Amaddeo G Baranes L. Multiphase liver MRI for identifying the macrotrabecular-massive subtype of hepatocellular carcinoma. Radiology (2020) 295:562–71. doi: 10.1148/radiol.2020192230 32228294

[12] Feng Z Li H Zhao H Jiang Y Liu Q Chen Q. Preoperative CT for characterization of aggressive macrotrabecular-massive subtype and vessels that encapsulate tumor clusters pattern in hepatocellular carcinoma. Radiology (2021) 300:219–29. doi: 10.1148/radiol.2021203614 33973839

[13] Zhu Y Weng S Li Y Yan C Ye R Wen L. A radiomics nomogram based on contrast-enhanced MRI for preoperative prediction of macrotrabecular-massive hepatocellular carcinoma. Abdom Radiol (2021) 46:3139–48. doi: 10.1007/s00261-021-02989-x 33641018

[14] Chen J Xia C Duan T Cao L Jiang H Liu X. Macrotrabecular-massive hepatocellular carcinoma: imaging identification and prediction based on gadoxetic acid–enhanced magnetic resonance imaging. Eur Radiol (2021) 31:7696–704. doi: 10.1007/s00330-021-07898-7 33856520

[15] Zhang J Wang X Zhang L Yao L Xue X Zhang S. Radiomics predict postoperative survival of patients with primary liver cancer with different pathological types. Ann Transl Med (2020) 8:820. doi: 10.21037/atm-19-4668 32793665 PMC7396247

[16] Verna EC Patel YA Aggarwal A Desai AP Frenette C Pillai AA. Liver transplantation for hepatocellular carcinoma: Management after the transplant. Am J Transplant (2020) 20:333–47. doi: 10.1111/ajt.15697 31710773

[17] Cao L Chen J Duan T Wang M Jiang H Wei Y. Diffusion kurtosis imaging (DKI) of hepatocellular carcinoma: correlation with microvascular invasion and histologic grade. Quant Imaging Med Surg (2019) 9:59002–602. doi: 10.21037/qims.2019.02.14 PMC6511714 31143650

[18] Consul N Sirlin CB Chernyak V Fetzer DT Masch WR Arora SS. Imaging features at the periphery: Hemodynamics, pathophysiology, and effect on LI-RADS categorization. Radiogr Rev Publ Radiol Soc N Am Inc (2021) 41:1657–75. doi: 10.1148/rg.2021210019 34559586

[19] Nitta H Allard M-A Sebagh M Ciacio O Pittau G Vibert E. Prognostic value and prediction of extratumoral microvascular invasion for hepatocellular carcinoma. Ann Surg Oncol (2019) 26:2568–76. doi: 10.1245/s10434-019-07365-0 31054040

[20] Ham JH Yu J-S Choi JM Cho E-S Kim JH Chung J-J. Corona enhancement can substitute enhancing capsule in the imaging diagnosis of small (≤ 3 cm) HCCs on gadoxetic acid-enhanced MRI. Eur Radiol (2021) 31:8628–37. doi: 10.1007/s00330-021-07911-z 33891153

[21] Cerny M Chernyak V Olivié D Billiard J-S Murphy-Lavallée J Kielar AZ. LI-RADS version 2018 ancillary features at MRI. Radiogr Rev Publ Radiol Soc N Am Inc (2018) 38:1973–2001. doi: 10.1148/rg.2018180052 30289735

[22] Wei H Jiang H Liu X Qin Y Zheng T Liu S. Can LI-RADS imaging features at gadoxetic acid-enhanced MRI predict aggressive features on pathology of single hepatocellular carcinoma? Eur J Radiol (2020) 132:109312. doi: 10.1016/j.ejrad.2020.109312 33022551

[23] Calderaro J Meunier L Nguyen CT Boubaya M Caruso S Luciani A. ESM1 as a marker of macrotrabecular-massive hepatocellular carcinoma. Clin Cancer Res Off J Am Assoc Cancer Res (2019) 25:5859–65. doi: 10.1158/1078-0432.CCR-19-0859 31358545

[24] Chan AWH Zhong J Berhane S Toyoda H Cucchetti A Shi K. Development of pre and post-operative models to predict early recurrence of hepatocellular carcinoma after surgical resection. J Hepatol (2018) 69:1284–93. doi: 10.1016/j.jhep.2018.08.027 30236834

[25] Lee S Kim SH Lee JE Sinn DH Park CK. Preoperative gadoxetic acid–enhanced MRI for predicting microvascular invasion in patients with single hepatocellular carcinoma. J Hepatol (2017) 67:526–34. doi: 10.1016/j.jhep.2017.04.024 28483680

[26] Cannella R Dioguardi Burgio M Beaufrère A Trapani L Paradis V Hobeika C. Imaging features of histological subtypes of hepatocellular carcinoma: Implication for LI-RADS. JHEP Rep Innov Hepatol (2021) 3:100380. doi: 10.1016/j.jhepr.2021.100380 PMC8603197 34825155

[27] Husain A Chiu Y-T Sze KM-F Ho DW-H Tsui Y-M Suarez EMS. Ephrin-A3/EphA2 axis regulates cellular metabolic plasticity to enhance cancer stemness in hypoxic hepatocellular carcinoma. J Hepatol (2022) S0168-8278(22):00125–8. doi: 10.1016/j.jhep.2022.02.018 35227773

[28] Ridder DA Weinmann A Schindeldecker M Urbansky LL Berndt K Gerber TS. Comprehensive clinicopathologic study of alpha fetoprotein-expression in a large cohort of patients with hepatocellular carcinoma. Int J Cancer (2022) 150:1053–66. doi: 10.1002/ijc.33898 34894400

[29] van Meer S de Man RA Coenraad MJ Sprengers D van Nieuwkerk KMJ Klümpen H-J. Surveillance for hepatocellular carcinoma is associated with increased survival: Results from a large cohort in the Netherlands. J Hepatol (2015) 63:1156–63. doi: 10.1016/j.jhep.2015.06.012 26100498

[30] Kang H-J Kim H Lee DH Hur BY Hwang YJ Suh K-S. Gadoxetate-enhanced MRI features of proliferative hepatocellular carcinoma are prognostic after surgery. Radiology (2021) 300:572–82. doi: 10.1148/radiol.2021204352 34227881

[31] Liang Y Xu F Wang Z Tan C Zhang N Wei X. A gadoxetic acid-enhanced MRI-based multivariable model using LI-RADS v2018 and other imaging features for preoperative prediction of macrotrabecular-massive hepatocellular carcinoma. Eur J Radiol (2022) 153:110356. doi: 10.1016/j.ejrad.2022.110356 35623312

