# Supplementary material for: Corona enhancement combined with microvascular invasion for prognosis prediction of macrotrabecular-massive hepatocellular carcinoma subtype
Source: Front Oncol. 2023 Feb 20;13:1138848. doi: 10.3389/fonc.2023.1138848 (PMC9986746; doi:10.3389/fonc.2023.1138848)
Supplement: Supplementary Table 1 — Clinical and Pathologic Features of Patients with Hepatocellular Carcinoma in the Primary and Validation Cohort. Note: Variables are expressed as median (interquartile range) or number of patients(percentage). Abbreviations: HBV= Hepatitis B virus, AFP= Alpha-fetoprotein, CEA= Carcinoembryonic antigen, CA125= Carbohydrate antigens 125, CA199= Carbohydrate antigens 19-9, SF= serum ferritin. * Available number of Carbohydrate antigens 125, Carbohydrate antigens 19-9 and Serum ferritin is 121, 122 and 121, independently. [file Table_1.docx]

| **Supplemental Table 1. Clinical and Pathologic Features of Patients with Hepatocellular Carcinoma in the Primary and Validation Cohort** | | | |  |
| --- | --- | --- | --- | --- |
|  |  |  |  |  |
| Variables | Primary Cohort | Validation Cohort | *P* |  |
|  | (n=123) | (n=59) |  |  |
| Age, years | 61 (50-66) | 59 (51-67) | 0.756 |  |
| Sex, male/female | 101/22 | 50/9 | 0.658 |  |
| HBV infection | 97 (78.9) | 44 (74.6) | 0.517 |  |
| AFP, ng/ml | 32.7 (4.10-389.1) | 20.1 (3.6-358.7) | 0.806 |  |
| CEA, ng/ml | 2.7 (1.8-4.0) | 2.4 (1.4-3.1) | **0.012** |  |
| ^*^CA125, U/ml | 11.6 (8.45-16.9) | 12.0 (8.0-16.8) | 0.349 |  |
| ^*^CA199, U/ml | 6.0 (3.0-14.0) | 8.9 (5.1-17.5) | **0.016** |  |
| ^*^SF, ng/ml | 218.6 (126.9-325.8) | 207.3 (122.9-324.1) | 0.931 |  |
| multiple tumor number | 10 (8.1) | 4 (6.8) | 0.749 |  |
| main tumor size (cm) | 3.5 (2.2-5.5) | 5.5 (4.2-7.2) | **<0.001** |  |
| Macrovascular invasion | 13 (10.6) | 8 (13.6) | 0.555 |  |
| Absent or incomplete capsule | 57 (46.3) | 20 (33.9) | 0.112 |  |
| MVI risk |  |  | 0.153 |  |
| 0 | 78 (63.4) | 43 (72.9) |  |  |
| 1 | 33 (26.8) | 14 (23.7) |  |  |
| 2 | 12 (9.7) | 2 (3.4) |  |  |
| Cirrhosis | 57 (46.3) | 20 (33.9) | **0.001** |  |
| MTM subtype | 53 (43.1) | 16 (27.1) | **0.038** |  |
|  |  |  |  |  |
| Note: Variables are expressed as median (interquartile range) or number of patients(percentage). Abbreviations: HBV= Hepatitis B virus, AFP= Alpha-fetoprotein, CEA= Carcinoembryonic antigen, CA125= Carbohydrate antigens 125, CA199= Carbohydrate antigens 19-9, SF= serum ferritin. | | | |  |
|  |  |  |  |  |
|  |  |  |  |  |
|  |  |  |  |  |
| ^*^ Available number of Carbohydrate antigens 125, Carbohydrate antigens 19-9 and Serum ferritin is 121, 122 and 121, independently. | | | |  |
|  |  |  |  |  |
