# Supplementary figures and images for: Corona enhancement combined with microvascular invasion for prognosis prediction of macrotrabecular-massive hepatocellular carcinoma subtype
Source: Front Oncol. 2023 Feb 20;13:1138848. doi: 10.3389/fonc.2023.1138848 (PMC9986746; doi:10.3389/fonc.2023.1138848)

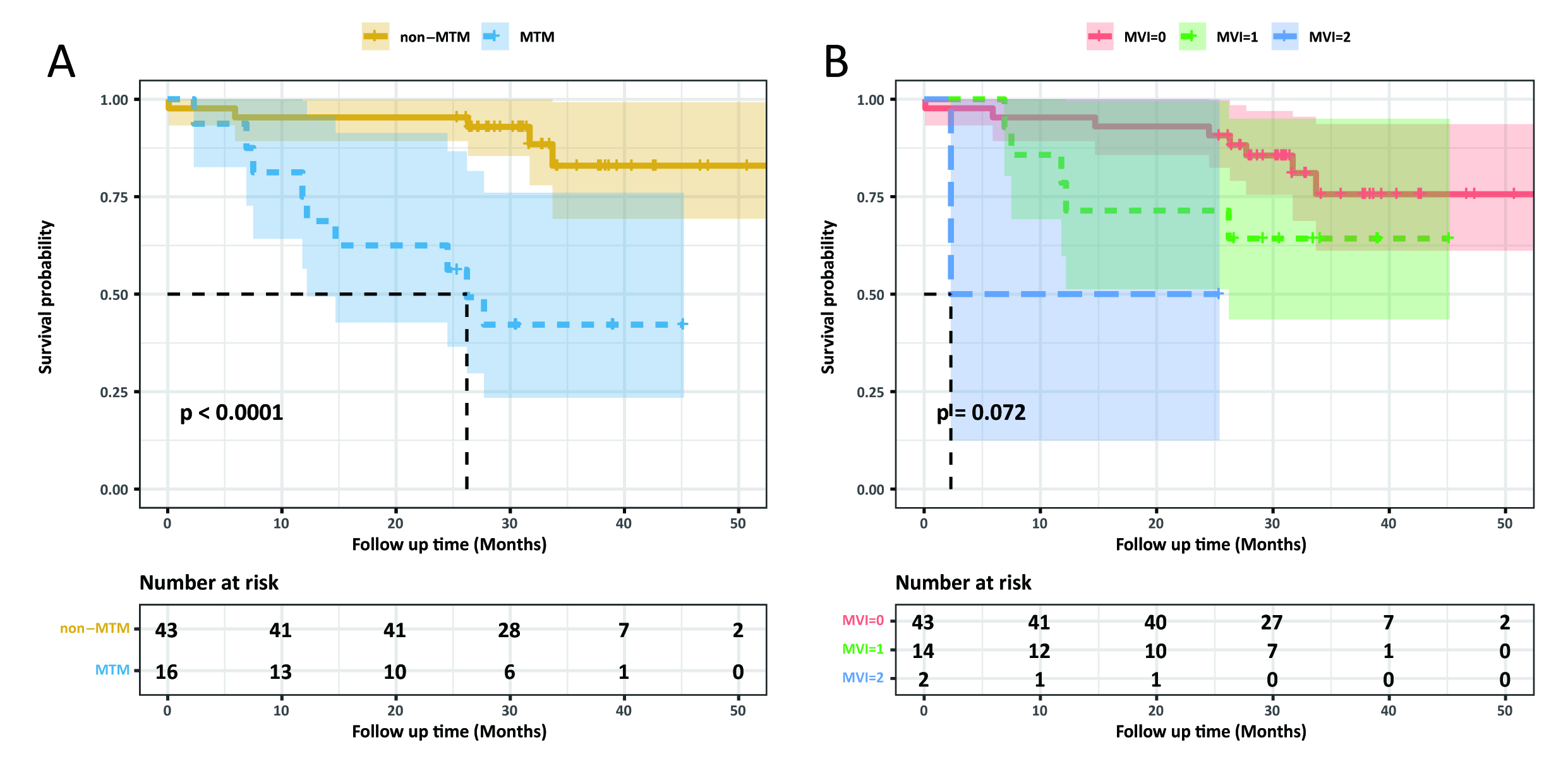

Supplement: Supplementary Figure 1 — Kaplan–Meier overall survival curves of MTM HCC and non-MTM HCC patients(A); different subgroups of MVI risk patients(B). [file Image_1.tif]
