# Supplementary material for: Corona enhancement combined with microvascular invasion for prognosis prediction of macrotrabecular-massive hepatocellular carcinoma subtype
Source: Front Oncol. 2023 Feb 20;13:1138848. doi: 10.3389/fonc.2023.1138848 (PMC9986746; doi:10.3389/fonc.2023.1138848)
Supplement: Supplementary file 3 [file Presentation_1.pdf]

Appendix 1

The MRI protocol

1) GE Discovery MR750: an axial T2WI fat-suppressed turbo-spin-echo sequence was performed with the following parameters: repetition time/echo time: 7058 ms/87 ms; field-of-view (FOV): 380 mm×380 mm; seam thickness: 5 mm. The breath-hold InPhase and OutPhase T1-weighted sequences were performed with the following parameters: repetition time/echo time: 200 ms/2.2 ms and 200 ms/1.1 ms. Diffusion-weighted imaging (DWI) was performed with the following parameters: repetition time/echo time: 9000 ms/60 ms; seam thickness: 5-6 mm; spacing: 1 cm; and diffusion coefficient b value: 1000 s/mm^2. The breath-hold T1WI sequence was performed with the following parameters: repetition time/echo time: 3.3 ms/1.2 ms; FOV: 328 mm×350 mm; reverse angle: 10°; seam thickness: 2 mm.

2) GE Signa HDxt: axial T2-weighted respiratory gating fast spin-echo sequences were performed with the following parameters: repetition time/echo time: respiratory rate related individual time /85 ms; FOV: 380 mm×285 mm; seam thickness: 5 mm. The breath-hold T1WI pressure-sensitive LAVA scan sequence was performed with the following parameters: repetition time/echo time: 3.1/1.5 ms; matrix: 320×256; layer thickness: 5 mm; and FOV: 400×320 mm. The DWI was performed with the following parameters: repetition time/echo time: 9000 mess/60.5 ms; FOV: 380 mm×285 mm; layer thickness: 5 mm; and diffusion coefficient b value: 1000 s/mm^2.
